# Ovarian Ectopic Pregnancy: A Case Report of Two Cases Highlighting Diagnostic and Management Challenges

**DOI:** 10.7759/cureus.92159

**Published:** 2025-09-12

**Authors:** Pradnya More, Megha Mishra, Sahar Mohamed

**Affiliations:** 1 Department of Obstetrics and Gynecology, Southend University Hospital, Southend-on-Sea, GBR

**Keywords:** corpus luteum hemorrhage, diagnostic challenges, ectopic pregnancy, hemoperitoneum, laparoscopy, miscarriage, ovarian ectopic pregnancy, pregnancy of unknown location, transvaginal ultrasound

## Abstract

Ovarian ectopic pregnancy (OEP) is a rare but potentially life-threatening form of ectopic gestation. Although recognized as a distinct clinical entity, its pathophysiology, diagnosis, and management remain poorly defined. Clinical presentation is often nonspecific and may mimic acute pelvic pathologies such as ruptured corpus luteum, hemorrhagic ovarian cysts, or miscarriage in women presenting with vaginal bleeding. In some cases, it resembles a pregnancy of unknown location (PUL) when the gestation cannot be localized on imaging, complicating preoperative diagnosis. Prompt recognition is critical to avoid severe hemorrhagic complications, and laparoscopy remains the mainstay of both diagnosis and treatment.

We describe two cases with distinct clinical presentations: one involving a 34-year-old multiparous woman presenting with acute pelvic pain, vaginal bleeding, hemoperitoneum, and a hemorrhagic right ovarian lesion confirmed histologically as OEP, and another a 17-year-old nulliparous woman initially managed as a PUL who later presented with syncope, nausea, and vomiting, where laparoscopy revealed a bleeding ovarian mass, which is also confirmed histologically as OEP.

OEP should be considered in reproductive-aged women presenting with acute abdominal pain, hemoperitoneum, and elevated beta human chorionic gonadotropin, even when ultrasound findings are inconclusive. These cases emphasize the need for a high index of suspicion and the role of laparoscopy in diagnosis and management. Early surgical intervention reduces morbidity and preserves ovarian function. Given its rarity and the absence of standardized guidelines, appropriate counseling is essential, and larger datasets are needed to inform future diagnostic and management protocols.

## Introduction

Ectopic pregnancy accounts for approximately 1%-2% of all pregnancies and remains a leading cause of maternal morbidity and mortality in the first trimester. Among its various forms, ovarian ectopic pregnancy (OEP) is one of the rarest subtypes, representing an estimated 0.5%-3.5% or approximately one in 7,000-40,000 live births of all ectopic gestations [1,2]. While traditionally considered uncommon, the reported incidence of OEP appears to be increasing, likely due to improved imaging techniques and heightened clinical awareness [3].

Although potentially life-threatening, the pathophysiology of OEP remains poorly understood. Diagnosis is particularly challenging, as the clinical presentation often mimics other acute adnexal conditions such as ruptured ovarian cysts, tubal ectopic, or hemorrhagic corpus luteal cysts. In many cases, clinical examination, serum beta human chorionic gonadotropin (β-hCG) levels, and imaging modalities like ultrasound or MRI lack sufficient diagnostic sensitivity and specificity, which can result in both missed and misdiagnosed cases, sometimes influenced by clinician bias [4,5].

While transvaginal ultrasound (TVS) with color Doppler can occasionally identify OEP preoperatively, laparoscopy remains the gold standard for both diagnosis and treatment. Intraoperative findings may still be inconclusive, and definitive diagnosis is often established postoperatively using the modified Spiegelberg criteria described by Wang et al., which include: 1) absence of pathological evidence of ipsilateral fallopian tube involvement and 2) demonstration of gestational tissue within the ovary, such as chorionic villi and/or an implantation site. If both criteria are satisfied, a diagnosis of primary ovarian pregnancy should be made [6]. There is currently no consensus on optimal management, with variability in surgical approaches and limited discussion around ovarian-sparing techniques. Posttreatment follow-up protocols are also not well established in current clinical guidelines.

We present two cases of OEP with markedly different clinical presentations. Both were managed with diagnostic laparoscopy and confirmed on histopathological examination. These cases illustrate the diagnostic challenges associated with OEP, emphasize the need for early surgical intervention, and highlight the importance of developing more standardized diagnostic and management protocols.

## Case presentation

Case 1

A 34-year-old woman with two previous vaginal deliveries presented with a seven-week history of intermittent vaginal bleeding that had worsened over the preceding 24 hours. She reported the passage of clots accompanied by left-sided pelvic pain radiating to the back, legs, and left shoulder tip. A urine pregnancy test was positive. She was a known smoker and had no significant past medical or surgical history.

On examination, she was hemodynamically stable. Abdominal palpation revealed tenderness in the left iliac fossa. Speculum examination showed mild vaginal spotting, while bimanual examination revealed a uterus of approximately six to eight weeks' size with marked left adnexal tenderness.

**Figure 1:**
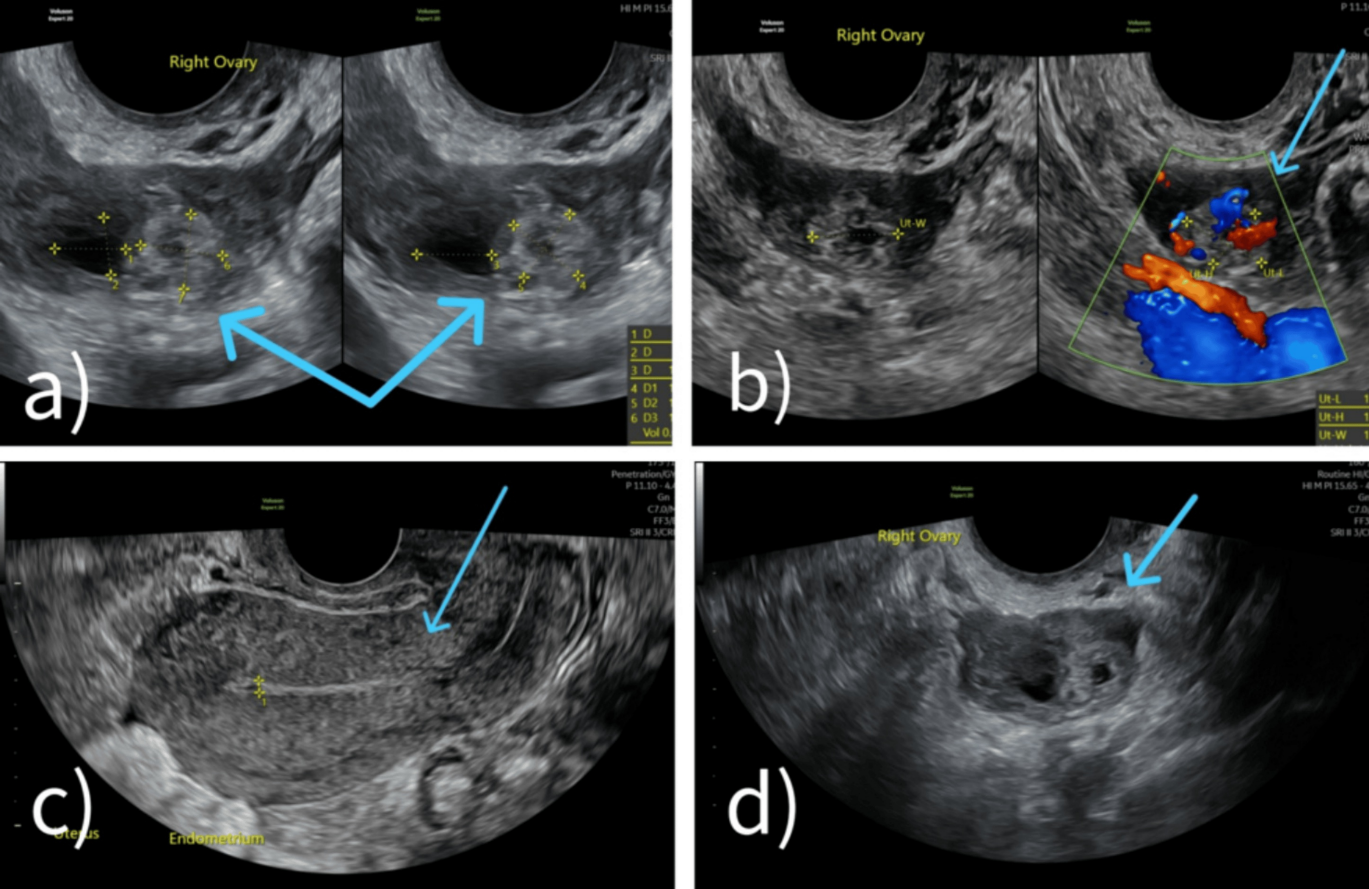
Transvaginal ultrasound images of Case 1 (a) Right ovary, where the double arrow indicates the cystic area suspicious for ectopic implantation vs. corpus luteum. (b) Right ovary with color Doppler showing vascular flow around the lesion, also referred to as the ring of fire sign. (c) Longitudinal view of the uterus with thin endometrium and no intrauterine gestational sac. (d) Right ovary, where the arrow points to a complex cystic mass with mixed echogenicity, consistent with either a hemorrhagic corpus luteum and/or ovarian ectopic tissue

A TVS scan was arranged due to high clinical suspicion of an ectopic pregnancy. The scan revealed an anteverted uterus (95 × 43 × 57 mm) with a thin endometrial lining (2.5 mm) and no intrauterine gestational sac. The right ovary showed a collapsed corpus luteum and a second echogenic area with an anechoic center and peripheral vascularity, measuring 14 × 10 × 14 mm. These findings raised suspicion for either a secondary corpus luteum or an ovarian ectopic pregnancy. A small trace of fluid was also seen exiting the cervix, consistent with ongoing bleeding (Figure 1).

Blood investigations showed a serum β-hCG level of 4,349 IU/L and a progesterone level of 7.6 nmol/L. Despite inconclusive imaging, the suspicion of ectopic pregnancy remained high. Considering a ruptured corpus luteal cyst, ovarian ectopic pregnancy, and miscarriage as possible differentials, a diagnostic laparoscopy was planned. The patient was counseled and consented to laparoscopy with the possibility of salpingectomy, excision of ovarian ectopic tissue, or salpingo-oophorectomy if required.

**Figure 2:**
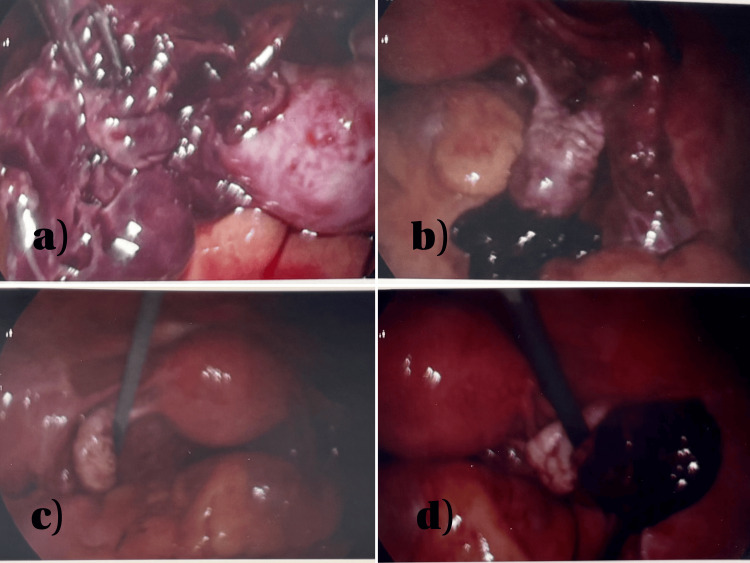
Intraoperative images (a) Intra-abdominal view showing the pregnancy tissue obtained. (b) Right ovary with hemorrhagic lesion, ectopic ovary, and ruptured corpus luteum cyst. (c) Normal uterus, normal left fallopian tube, and normal left ovary. (d) Right ovary with hemorrhagic lesion

Intraoperatively, the uterus, left ovary, and left fallopian tube appeared normal. The right fallopian tube was also unremarkable. However, the right ovary revealed a hemorrhagic area with ambiguous tissue. Approximately 200 mL of hemoperitoneum was evacuated, and the affected ovarian tissue was excised with hemostasis achieved by bipolar cauterization. The leading differential was a ruptured corpus luteum cyst, but the possibility of an ovarian ectopic pregnancy could not be excluded (Figure 2).

Repeat serum β-hCG the following day showed a decline to 1,837 IU/L. Histopathological analysis confirmed the presence of chorionic villi within the excised ovarian tissue, establishing the diagnosis of a ruptured ovarian ectopic pregnancy. On long-term follow-up, β-hCG returned to nonpregnant levels.

Case 2

A 17-year-old nulliparous woman with a positive urine pregnancy test was initially managed as a pregnancy of unknown location (PUL) following a one-day history of right iliac fossa pain. On clinical examination, she had localized tenderness in the right iliac fossa. Initial serum β-hCG was 265 IU/L, and serum progesterone was 14.2 nmol/L. A TVS at the time revealed no evidence of an intrauterine or extrauterine pregnancy. She was placed on expectant management with a plan for repeat β-hCG at 48 hours and a follow-up scan one week later.

At 48 hours, the β-hCG had risen to 559 IU/L. However, on the day of her scheduled follow-up scan a week later, the patient experienced a syncopal episode and vomiting at home and was brought to the emergency department. On presentation, she was hemodynamically stable, with a blood pressure of 103/61 mmHg and pulse of 84 bpm.

**Figure 3:**
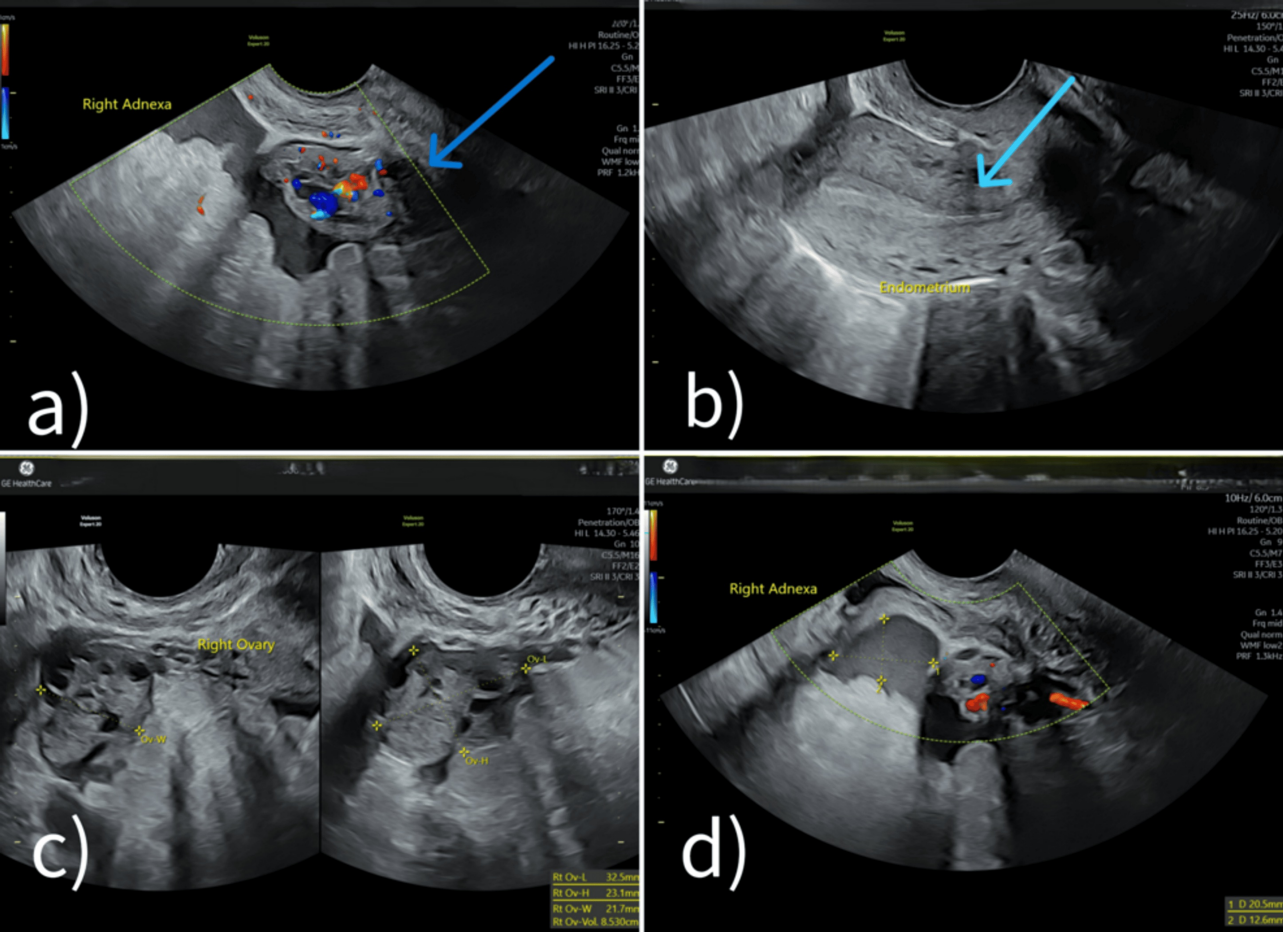
Transvaginal ultrasound images (a) Right adnexa with color Doppler showing a heterogeneous adnexal mass with internal vascularity. (b) Endometrium appears empty, without evidence of an intrauterine gestational sac. (c) Right ovary with the adnexal mass. (d) Right adnexa with color Doppler showing a well-defined, rounded mass with vascularity

A repeat TVS showed an anteverted uterus measuring 63 × 32 × 38 mm with an endometrial thickness of 5.1 mm. Both ovaries were visualized and appeared morphologically normal, but were extremely tender on probe pressure. Echogenic free fluid collections were identified in both adnexal regions, measuring 24 × 13 mm on the right and 24 × 19 mm on the left, raising a strong suspicion of a ruptured ectopic pregnancy (Figure 3).

**Figure 4:**
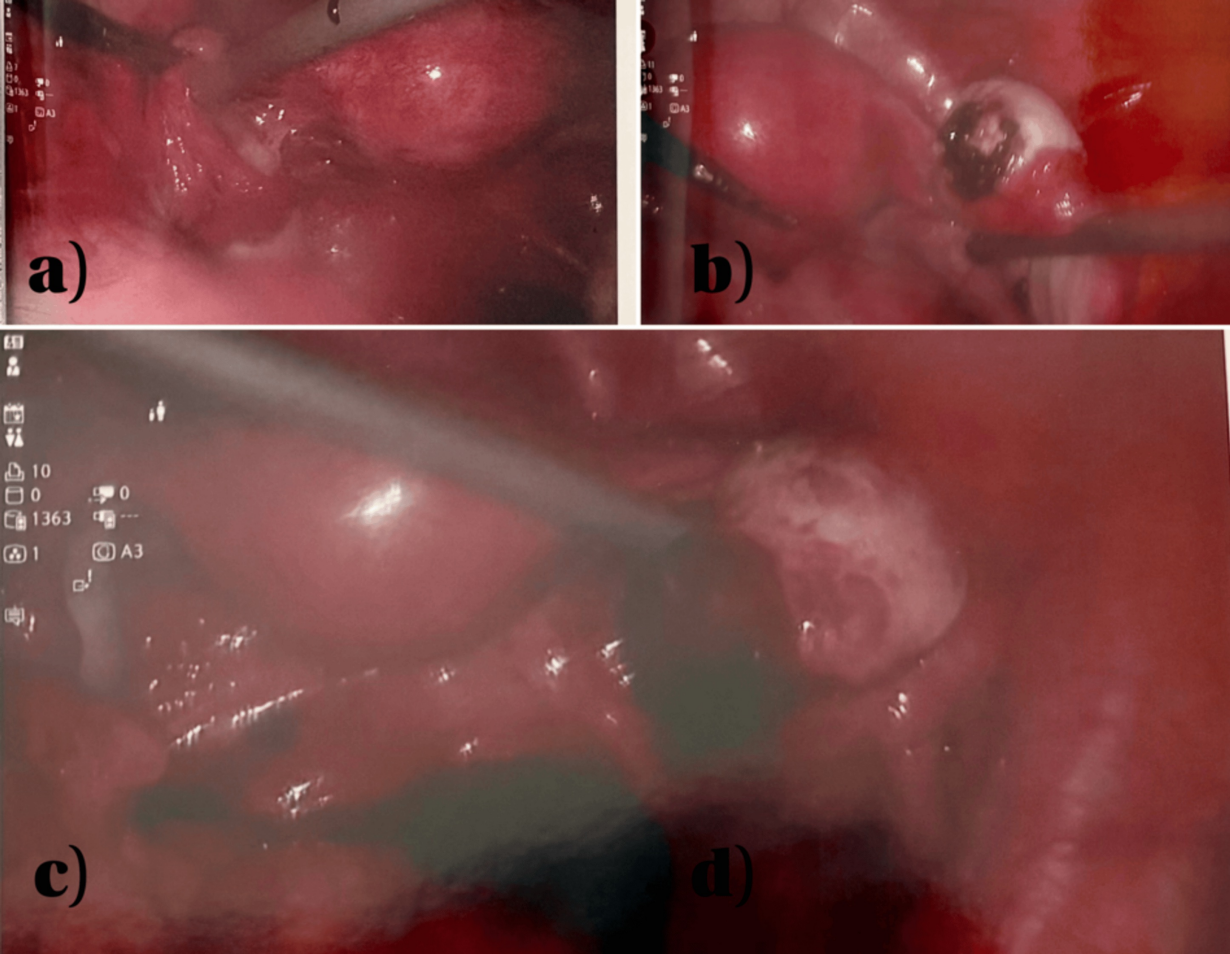
Intraoperative images of Case 2 (a) Intraoperative image of the uterus with a normal left tube and ovary. (b) Right ovary with hemorrhagic changes, posthemostasis with diathermy. (c) Another view of the right ovary showing an adnexal gestational sac-like structure, possibly ectopic

Given the clinical picture, a diagnostic laparoscopy was performed. Intraoperative findings included 100-150 mL of hemoperitoneum and a 1 × 1.5 cm actively bleeding mass on the right ovary. The uterus, both fallopian tubes, left ovary, appendix, and the remainder of the peritoneal cavity appeared normal. The right ovarian mass was excised using monopolar and bipolar diathermy, and hemostasis was achieved. The specimen was sent for histopathological examination (Figure 4).

On the day of surgery, serum β-hCG had increased to 6,780 IU/L. Postoperatively, β-hCG levels were rechecked at 48 hours and had dropped to 1,186 IU/L. Histopathological analysis confirmed the presence of chorionic villi in the excised tissue, consistent with a ruptured right ovarian ectopic pregnancy. Both fallopian tubes were confirmed to be uninvolved (Table 1).

**Table 1:** Serial β-hCG and progesterone levels in Cases 1 and 2 In early viable intrauterine pregnancy, serum β-hCG levels typically double approximately every 48 hours. A decline of >50% suggests a failing pregnancy, whereas a suboptimal rise, plateau, or inadequate fall may indicate an ectopic pregnancy. Progesterone levels are variable depending on the stage and outcome of pregnancy; no definitive cutoff exists, but a value <20 nmol/L most likely indicates a nonviable pregnancy β-hCG: beta human chorionic gonadotropin

Case	Investigation	Units	First presentation	48 hours later	Day of surgery	24 hours postop	48 hours postop	2 weeks postop	Reference range
1	β-hCG	IU/L	-	-	4,349	1,837	-	37	<5 (nonpregnant); doubles every 48 hours in viable intrauterine pregnancy
1	Progesterone	nmol/L	-	-	7.6	-	-	2.3	<20 nonviable pregnancy
2	β-hCG	IU/L	265	559	6,780	-	1,186	-	<5 (nonpregnant); doubles every 48 hours in viable intrauterine pregnancy
2	Progesterone	nmol/L	14.2	-	10.3	-	2.6	-	<20 nonviable pregnancy

## Discussion

OEP is an uncommon form of extrauterine gestation with significant diagnostic and therapeutic challenges. Its rarity, combined with nonspecific clinical and imaging findings, often leads to delayed or missed diagnosis, increasing the risk of morbidity.

Ovarian pregnancy accounts for approximately 0.5%-3% of all ectopic pregnancies and occurs in an estimated one in 7,000-40,000 live births [7]. Over a five-year period, we identified two cases of OEP out of a total of 365 ectopic pregnancies managed at our center, corresponding to an incidence of 0.54%.

Primary ovarian pregnancy refers to the direct implantation of the gestational sac within the ovarian tissue. In contrast, secondary ovarian pregnancy is thought to result from fertilization occurring in the fallopian tube, followed by retrograde migration of the conceptus into the ovarian stroma [8]. The first documented case of ovarian pregnancy was described in 1682 [9]. The mean gestational age at diagnosis is approximately seven weeks. Due to the high risk of early rupture, the majority of OEPs, around 91%, are diagnosed and managed during the first trimester. A smaller proportion progress beyond this point, with 5.4% continuing into the second trimester and only 3.7% reaching the third trimester [10,11].

The overall incidence of ectopic gestation has increased, likely due to the rising prevalence of sexually transmitted infections, pelvic inflammatory disease, the use of assisted reproductive technologies, and improved access to diagnostic facilities. The incidence of OEP in particular has been linked to intrauterine contraceptive device (IUCD) use. While IUCDs are effective in preventing intrauterine implantation, they do not confer protection against extrauterine pregnancies. It has been postulated that IUCDs may promote ovarian implantation by altering prostaglandin synthesis, thereby increasing tubal motility and facilitating the displacement of the fertilized ovum into ectopic locations, including the ovary [12]. This suggests that women using IUDs are more likely to develop an ovarian pregnancy than an intrauterine pregnancy, as the IUDs reduce the likelihood of intrauterine implantation but do not provide the same protective effect against ovarian implantation.

Recent literature quotes an increased incidence of OEP with infertility and assisted reproductive techniques. The incidence of ovarian pregnancy following in vitro fertilization embryo transfer (IVF-ET) is estimated at around 6% of all ectopic pregnancies, which is notably higher than the approximately 3% reported after natural conception [13]. Several mechanisms have been proposed to explain this increased incidence. One theory is reverse migration of a transferred embryo toward the fallopian tube, followed by implantation within the ovary. Lesny et al. demonstrated that a difficult embryo transfer can stimulate junctional zone contractions and that strong endometrial waves originating in the fundal region may propel embryos into the fallopian tubes [14].

The etiology of OEP is not fully understood, although it is most commonly postulated to result from reflux of the fertilized oocyte into the ovary. Other proposed mechanisms include interference with the release of the ovum from the ruptured follicle, dysfunction of the fallopian tubes, and inflammatory thickening of the ovarian tunica albuginea. Pathogenesis may involve fertilization occurring outside the fallopian tube, followed by implantation within the ovarian stroma. The ovary is covered by the tunica albuginea, a structure devoid of muscle fibers, with loose connective tissue and blood vessels within. This lack of muscular support may contribute to the tendency for early rupture. As the trophoblastic tissue invades the ovarian stroma, it disrupts surrounding blood vessels, leading to rapid accumulation of intra-abdominal blood once rupture occurs. This explains why ovarian pregnancies frequently present with hemoperitoneum and, in some cases, hemodynamic instability. In addition, the absence of decidualized endometrium within the ovary may limit the capacity of the tissue to accommodate implantation, further contributing to early rupture [15].

OEP presents with variable clinical features, often resembling those of a tubal ectopic pregnancy. Common symptoms include a period of amenorrhea, irregular vaginal bleeding, and lower abdominal pain. On examination, the uterus is usually of normal size, with adnexal tenderness, and, in some cases, a palpable adnexal mass. A proportion of patients may be asymptomatic. Abdominal examination may reveal tenderness, with or without guarding, indicating peritoneal irritation. In cases of rupture, patients may develop sudden, severe abdominal pain, and significant hemorrhage can lead to hypovolemic shock.

In 1878, Spiegelberg proposed the criteria for diagnosing ovarian pregnancy: 1) the ipsilateral tube must be intact, 2) the gestational sac must occupy a position in the ovary, 3) the ovary must be attached to the uterus through the utero-ovarian ligament, and 4) there must be ovarian tissue attached to the pregnancy in the specimen. These criteria continue to be the standard for the diagnosis of ovarian pregnancy at the time of surgery. They are useful to differentiate ovarian pregnancy from other types of ectopic pregnancy, but cannot be applied in ultrasound [16].

Despite improvements in modern ultrasound technology, identifying a ruptured OEP before surgery remains challenging. In fact, a definitive preoperative diagnosis is made in only 5.3%-25% of cases [17]. Ultrasonography may demonstrate a wide hyperechoic ring or mass caused by gestational trophoblastic tissue infiltrating the surrounding ovarian stroma, with echogenicity greater than that of a normal ovary or corpus luteum. Additional sonographic findings can include a complex adnexal mass, with or without free fluid in the pouch of Douglas, and ovarian enlargement. Differentiating a ruptured ovarian pregnancy from a ruptured tubal ectopic, hemorrhagic corpus luteum, or endometriotic (chocolate) cyst can be challenging due to their similar ultrasonographic appearances. Terzić et al. reported that in 75% of cases, a ruptured ovarian pregnancy is sonographically mistaken for a ruptured corpus luteum [18]. We encountered a similar diagnostic challenge in one of our cases, where the OEP was initially suspected to be a ruptured corpus luteum cyst.

According to a retrospective case-control study conducted at a single center over a 10-year period, which examined 20 women with OEP and compared them with 100 women with tubal ectopic pregnancy (TEP), key ultrasound features of OEP include localization of the gestational sac within the ovarian stroma and the inability to separate the pregnancy from the ovary on gentle probe palpation. The use of color Doppler was found to be valuable in demonstrating peritrophoblastic flow distinct from that of the corpus luteum; however, this finding is not entirely specific, as it may also be present in tubal pregnancies that are firmly adhered to the uterus or ovary [19]. The presence of a yolk sac or embryo within the ovarian cortex is highly specific of an ovarian pregnancy.

In both of our patients, serum β-high levels were elevated. Although TVS findings were inconclusive, there was a suspicion of OEP based on imaging, coupled with symptoms of severe abdominal pain and sonographic evidence of hemoperitoneum. These factors prompted urgent laparoscopy, which proved critical for timely diagnosis and intervention. However, diagnostic assessments such as an ultrasound may not always be definitive. Lee et al. reported a patient with OEP who presented at 38 weeks' gestation with decreased fetal movements; in this case, ultrasound failed to identify the OEP, instead showing a fetus in vertex presentation, and the gestational sac within the left ovary was only discovered intraoperatively [20]. While ultrasound plays a central role in the evaluation of ectopic pregnancies, a high index of suspicion remains essential, as it may fail to detect OEP in some cases. Routine prenatal assessments can facilitate earlier diagnosis and improve outcomes; nevertheless, some patients may remain undiagnosed despite appropriate antenatal care, adding to the complexity of diagnosis.

Ideally, management should be initiated prior to rupture of the OEP. Treatment options are broadly similar to those for other ectopic pregnancies, with the choice of approach guided by the patient's clinical presentation, reproductive wishes, and the treatment protocols available at the treating facility.

Surgical intervention serves both diagnostic and therapeutic purposes and is generally recommended as the first-line management option. The approach may involve laparoscopy or, in selected cases, laparotomy, with the primary objectives being diagnosis and removal of the ectopic pregnancy tissue, achievement of hemostasis, and preservation of as much healthy ovarian tissue as possible. Laparotomy is indicated as an emergency in patients presenting with hemodynamic instability, such as hypovolemic shock, or in the presence of significant hemoperitoneum.

Medical management of OEP remains a subject of debate, with limited evidence available in the literature. The main advantage is the preservation of ovarian tissue, making it a potential option for young women wishing to maintain fertility. Methotrexate may be considered if specific criteria are met: 1) absence of hemodynamic instability, 2) no sonographic evidence of pelvic free fluid, 3) a gestational mass measuring less than 3.5 cm without fetal cardiac activity, and 4) a serum β-high level below 3,500 IU/L [21].

## Conclusions

OEP is a rare but potentially life-threatening condition, and clinical awareness is essential to reduce associated morbidity and mortality. It should be considered in the differential diagnosis for women of reproductive age presenting with acute abdominal pain, even when hemodynamic parameters are stable. Risk factors such as current intrauterine device use and conception via IVF-ET may increase the likelihood of OEP, and affected patients often present with higher serum β-hCG levels and more severe clinical outcomes, including rupture, hemoperitoneum, and shock.

Diagnosis can be challenging due to nonspecific clinical features and overlapping imaging appearances with hemorrhagic ovarian cysts, bleeding corpus luteum, or tubal ectopic pregnancies. Sonographic interpretation may be further limited by the presence of a hematocele or hemoperitoneum. Laparoscopy remains the gold standard for both diagnosis and management, with intraoperative confirmation guided by Spiegelberg's criteria. Early recognition is critical to preserve fertility, avoid the need for emergency laparotomy, and improve clinical outcomes.
